# Supplementary material for: A teichoic acid-like wall modification associated with immune suppression is socially regulated in Streptococcus pyogenes
Source: mBio. 2026 Feb 24;17(4):e03801-25. doi: 10.1128/mbio.03801-25 (PMC13059722; doi:10.1128/mbio.03801-25)
Supplement: Supplemental material — Figures S1-S4 and Tables S1-S3. [file mbio.03801-25-s0001.pdf]

Supplemental Information

# A teichoic acid-like wall modification associated with immune suppression is socially regulated in *Streptococcus pyogenes*

Caleb M. Anderson, Reid V. Wilkening, Samy Boulos, Timothy G. Keys, Marc-Olivier Ebert, Léa V. Zinsli, Janes Krusche, Martin J. Loessner, Sam Feldstein , Jennifer C. Chang, Andreas Peschel, Alexander R. Horswill, Yang Shen, and Michael J. Federle.

## Contents

Figure S1. Rgg2/Rgg3 QS system depicting regulation of the *qim* operon Page 2
Figure S2. Mouse images, day 1 Page 3
Figure S3. Mouse images, day 3 Page 4
Figure S4. Mouse images, day 5 Page 5
Figure S5. Mouse images, day 7 Page 6
Figure S6. AlphaFold 2.0 predicted structures of *qim* operon proteins Page 7
Figure S7. NMR of purified carbohydrates Pages 8-9
Figure S8. UPLC-MS/MS monomer HF digestion analysis wildtype and Δqim Page 10
Table S1. Strains and plasmids used in this study Page 11
Table S2. Primers used in this study Page 12
Table S3. NMR Chemical shifts ($^{1}$H AND $^{13}$C) of Group A Carbohydrate Page 13

For high resolution version of all figures, see DOI: 10.6084/m9.figshare.30870698

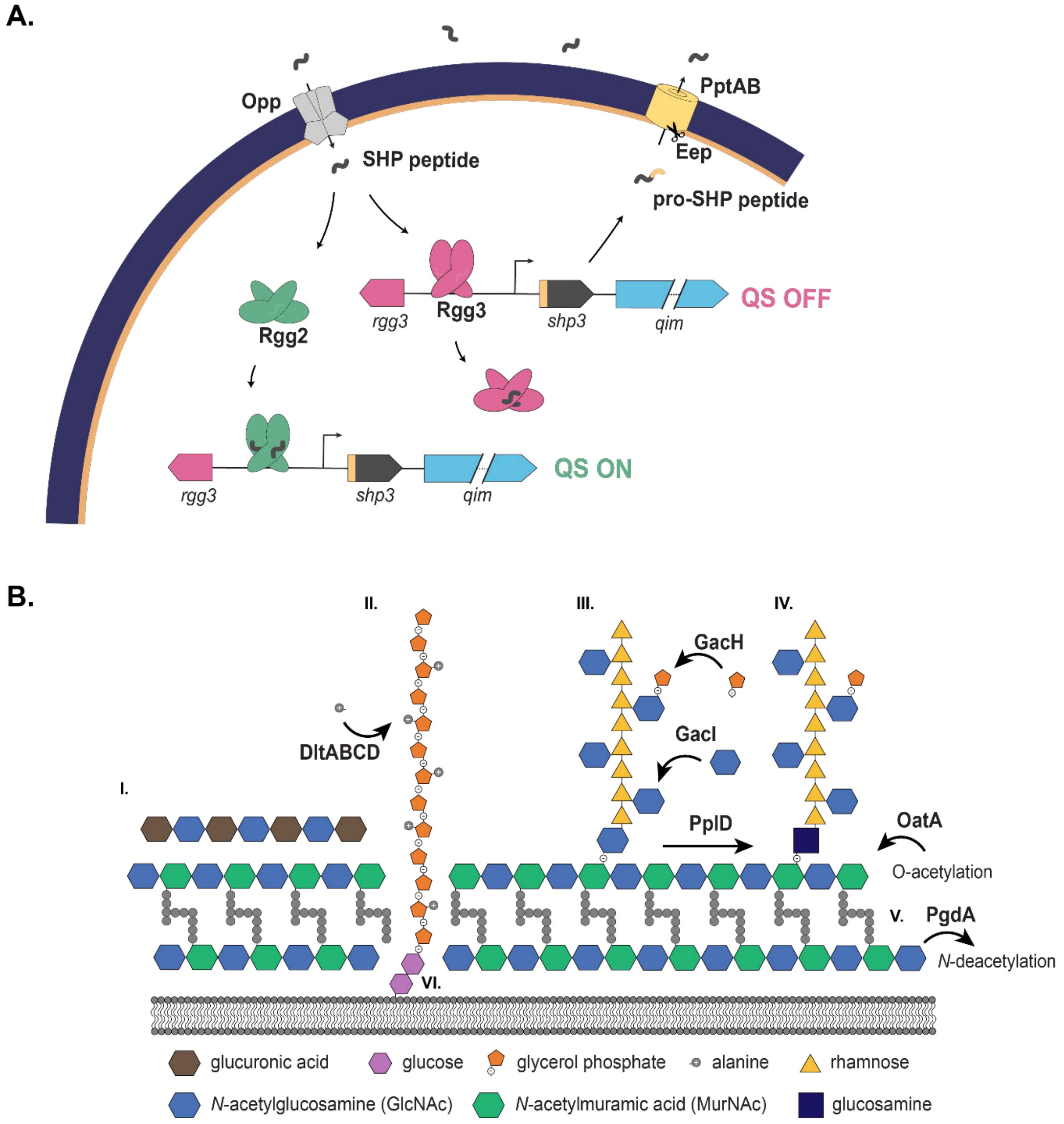


**Figure S1.** Rgg2/Rgg3 QS system depicting regulation of the *Spy49_0450-0460* (*qim*) operon. **(A)** In the QS-OFF state, in which SHP pheromone concentrations are low, the transcriptional regulator Rgg3 represses the QS system. When SHP enters the cell and binds to Rgg3, repression is relieved, while SHP binding to Rgg2 causes transcriptional activation of target operons. The resulting effect is further production of SHP, thus raising pheromone concentrations and activation of the QS system (QS-ON). **(B)** The major cell wall associated carbohydrates and polymers of *S. pyogenes*: hyaluronic acid capsule (I), lipoteichoic acid (II) anchored to the lipid membrane (VI), Group A Carbohydrate linked via *N*-acetylglucosamine phosphate (III) to peptidoglycan (V), and Group A Carbohydrate linked via glucosamine phosphate (IV) to peptidoglycan.

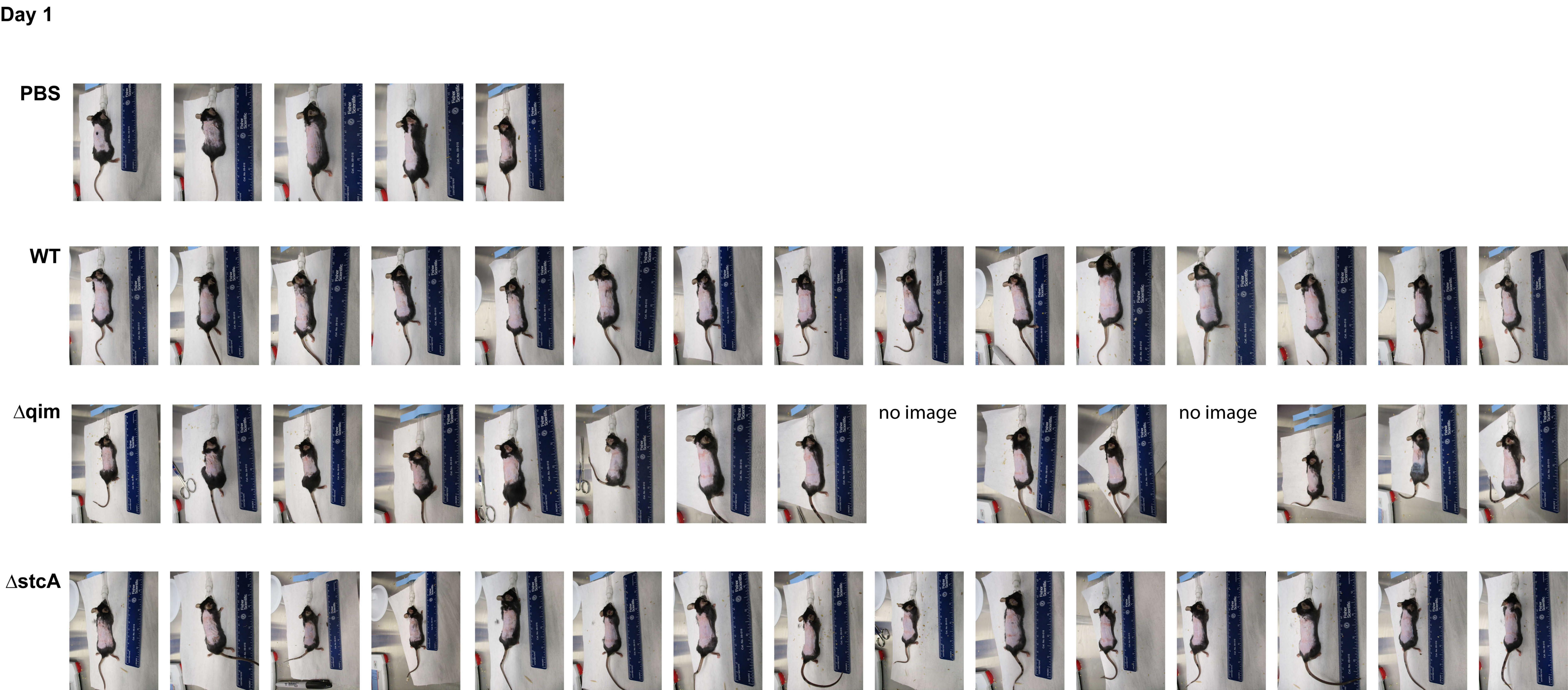
Day 1
PBS
WT
Δqim
no image
no image
ΔstcA

**Day 3**

**PBS**

**WT**

**Δqim**

**ΔstcA**

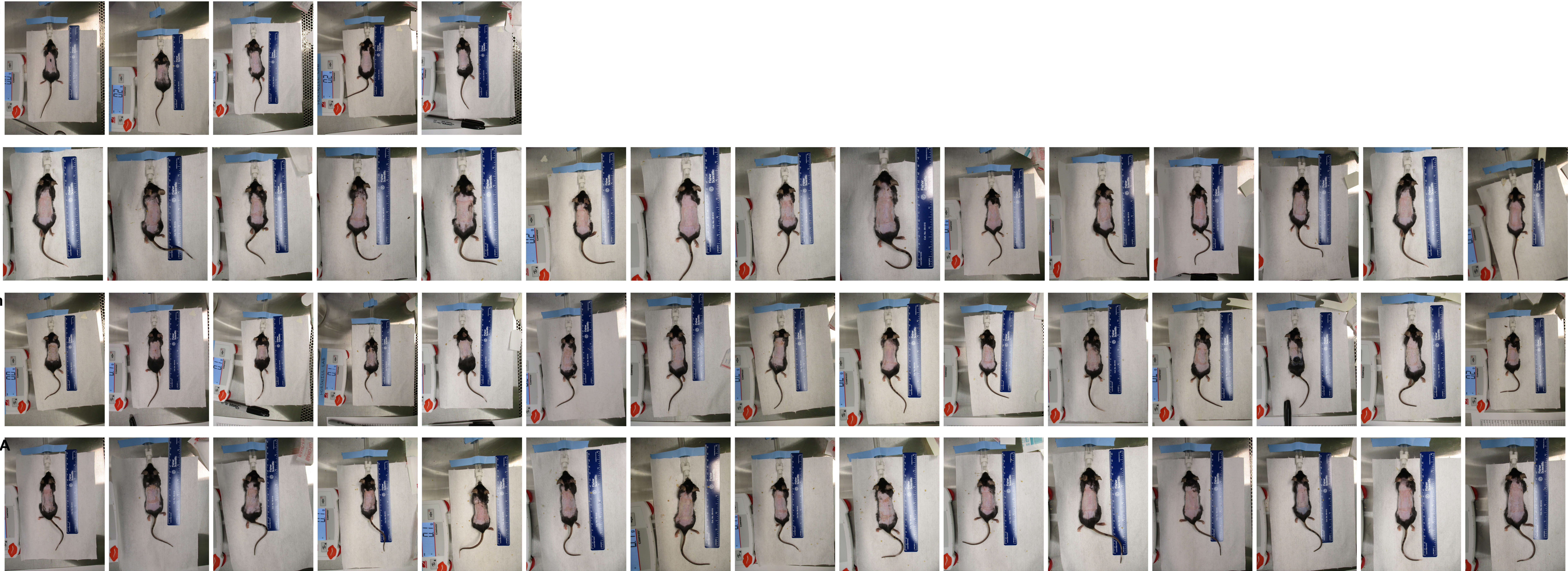

## Day 5

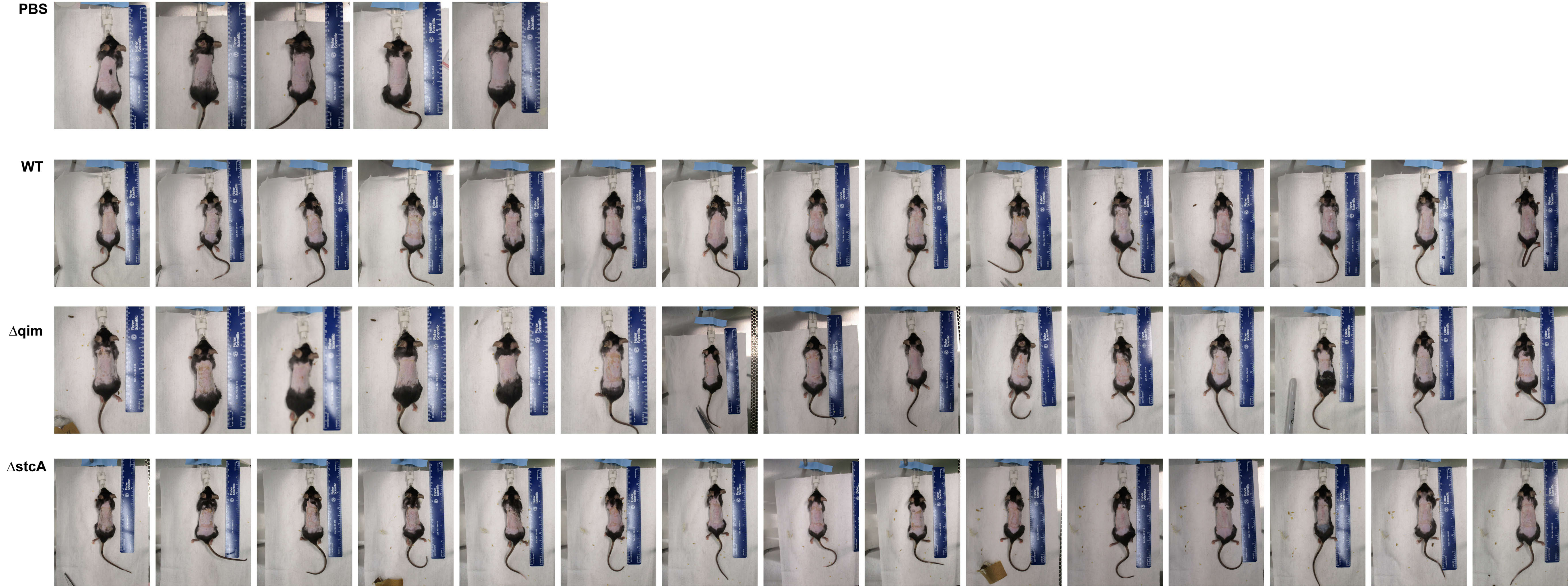

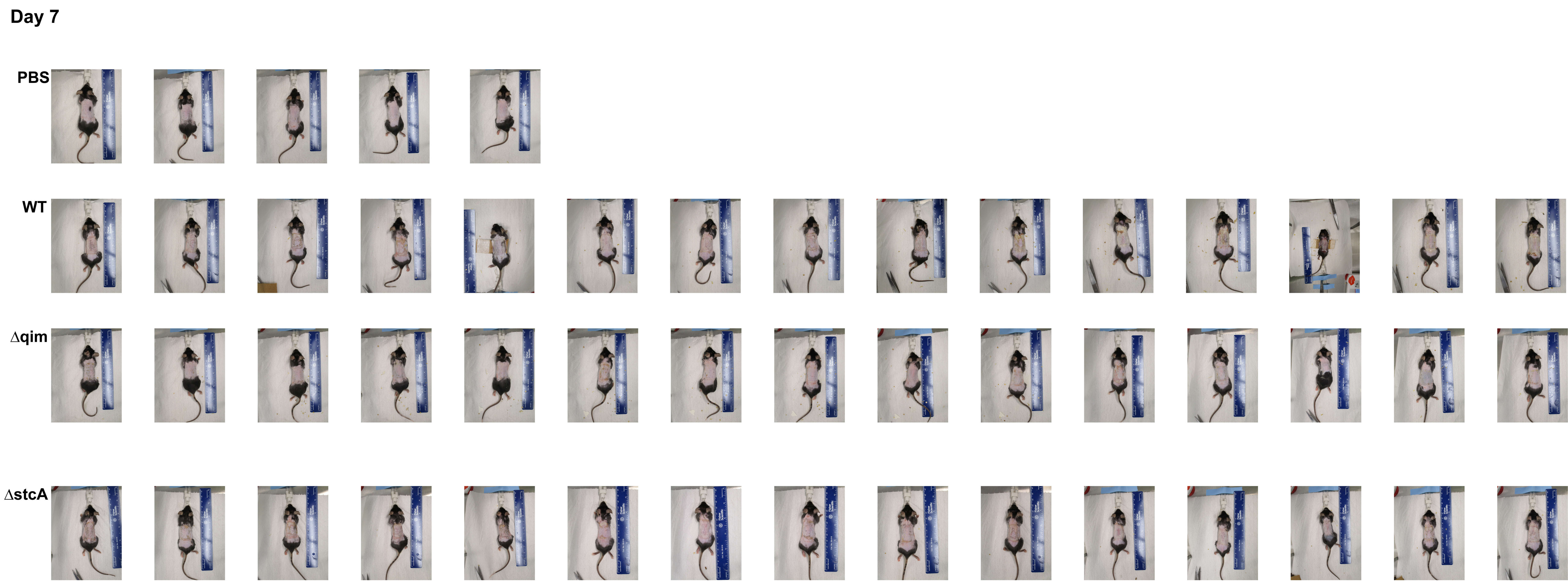
Day 7
PBS
WT
Δqim
ΔstcA

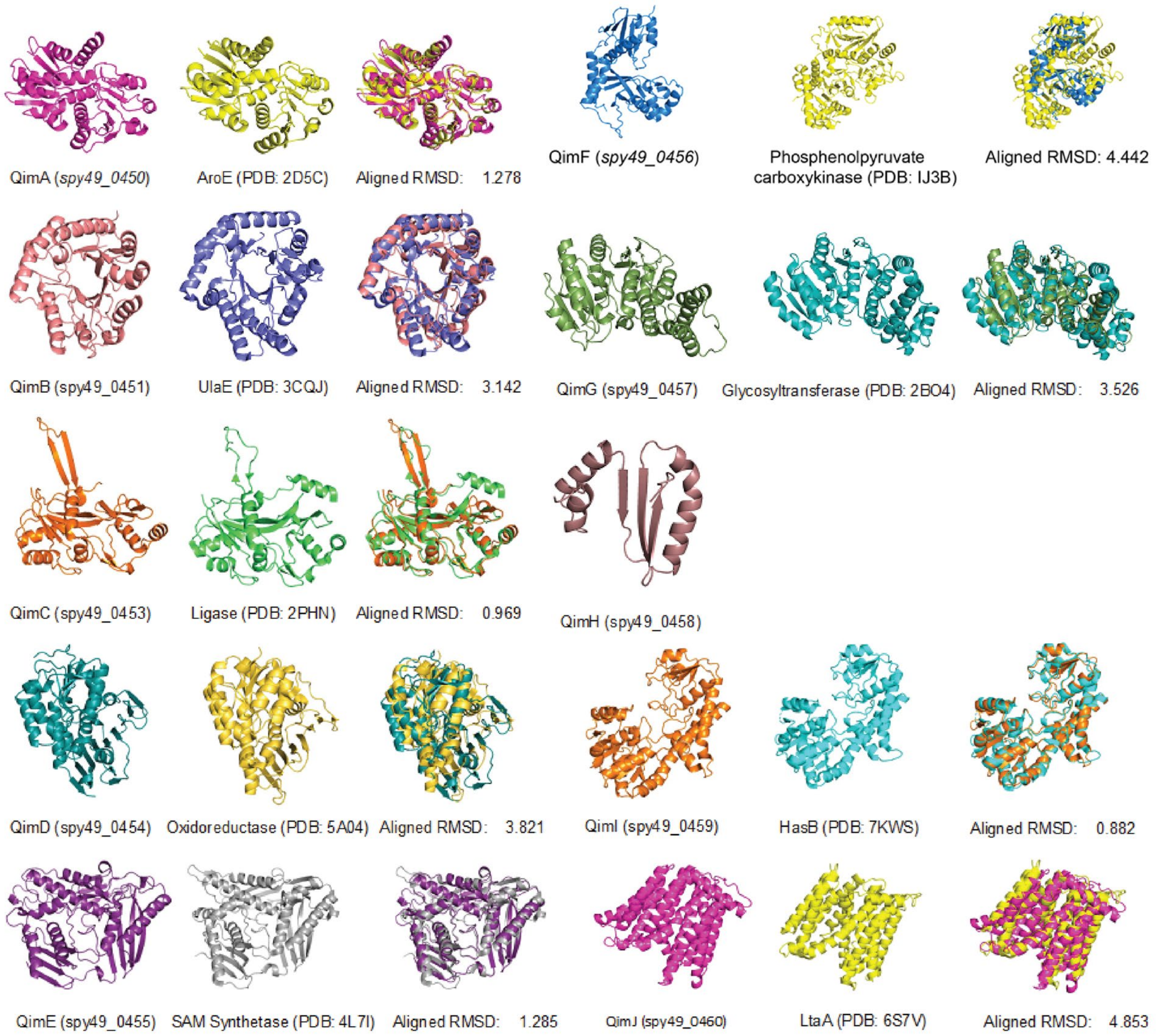


**Figure S6.** AlphaFold 2.0 predicted structures of *qim* operon proteins with matching HHpred hits and aligned structures showing the aligned RMSD.

A

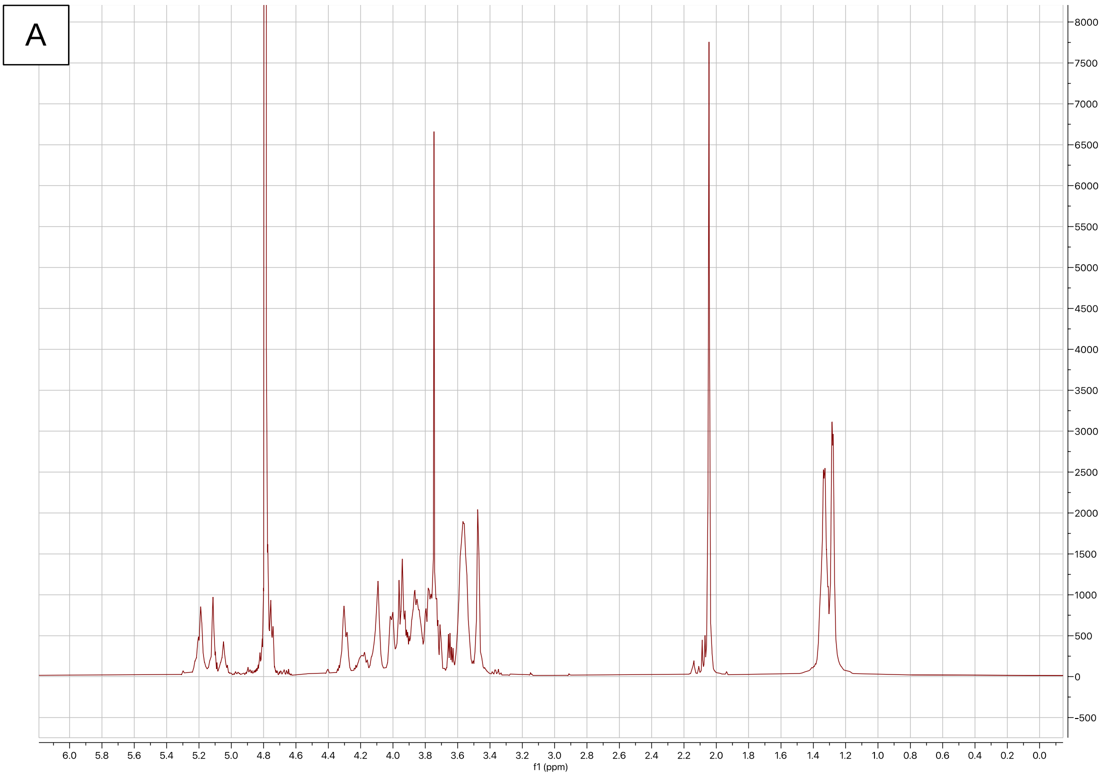

8000
7500
7000
6500
6000
5500
5000
4500
4000
3500
3000
2500
2000
1500
1000
500
0
-500
6.0 5.8 5.6 5.4 5.2 5.0 4.8 4.6 4.4 4.2 4.0 3.8 3.6 3.4 3.2 3.0 2.8 2.6 2.4 2.2 2.0 1.8 1.6 1.4 1.2 1.0 0.8 0.6 0.4 0.2 0.0
f1 (ppm)


B

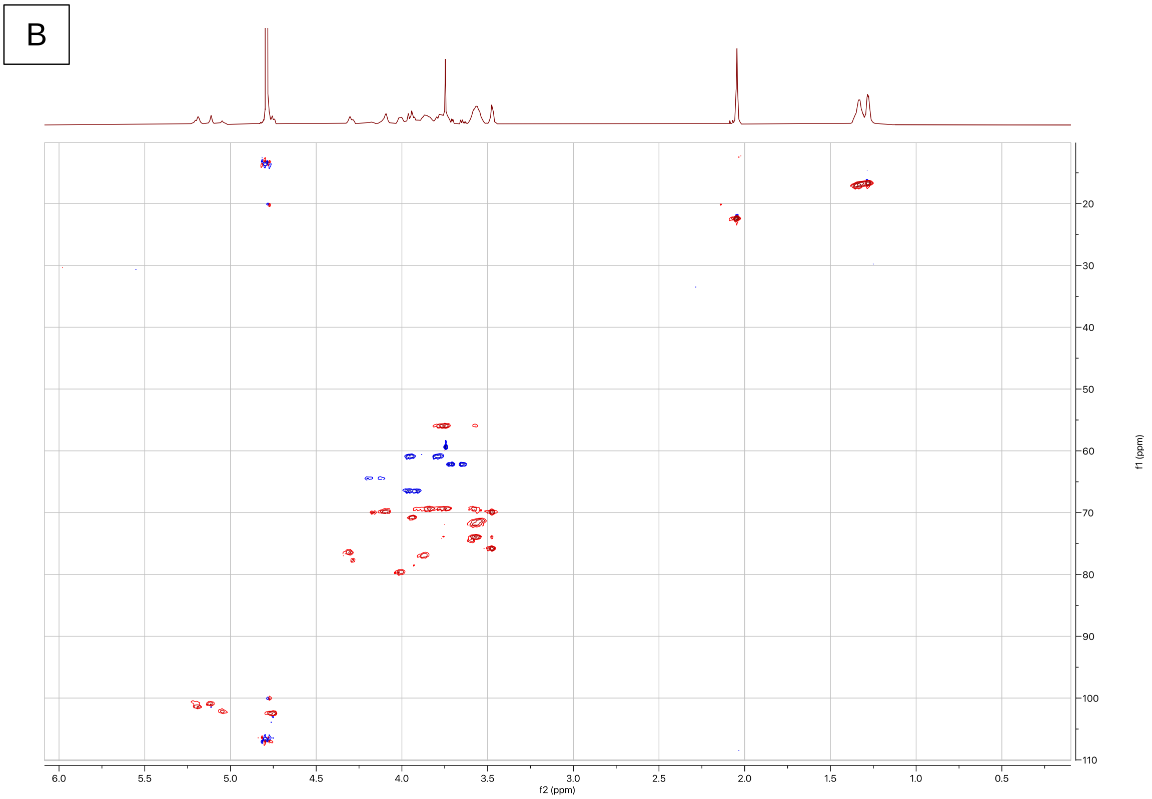

20
30
40
50
60
70
80
90
100
110
f1 (ppm)
6.0 5.5 5.0 4.5 4.0 3.5 3.0 2.5 2.0 1.5 1.0 0.5
f2 (ppm)

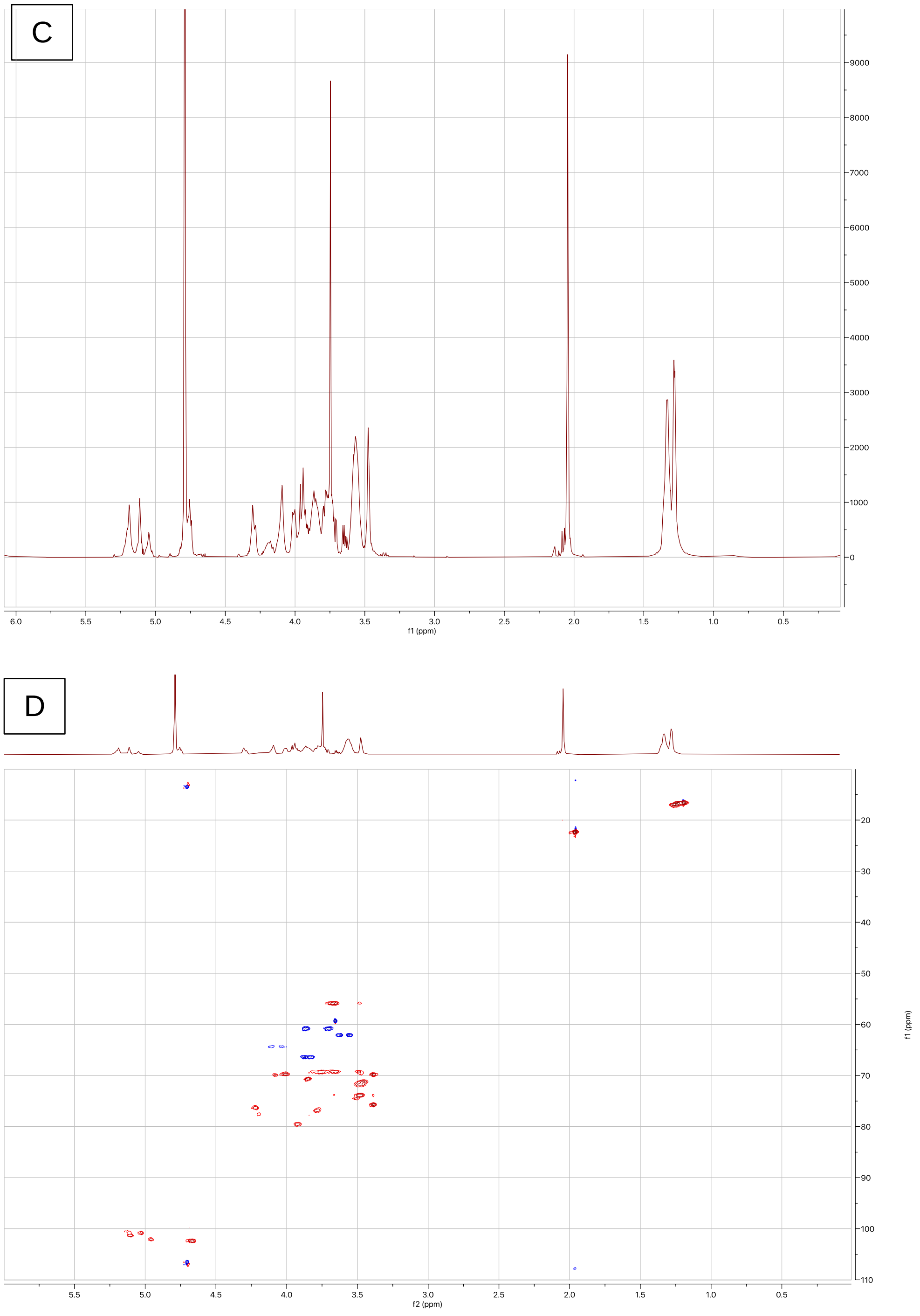


**Figure S7.** NMR of purified carbohydrates. (A) $^{1}$H NMR (GAC; GlcN-phosphate linked) – WT (QS-ON). (B) $^{1}$H/13C HSQC NMR (GAC; GlcN-phosphate linked) – WT (QS-ON). (C) $^{1}$H NMR (GAC; GlcN-phosphate linked) – Δ*qim* (QS-ON). (D) $^{1}$H/$^{13}$C HSQC NMR (GAC; GlcN-phosphate linked) – Δ*qim* (QS-ON).

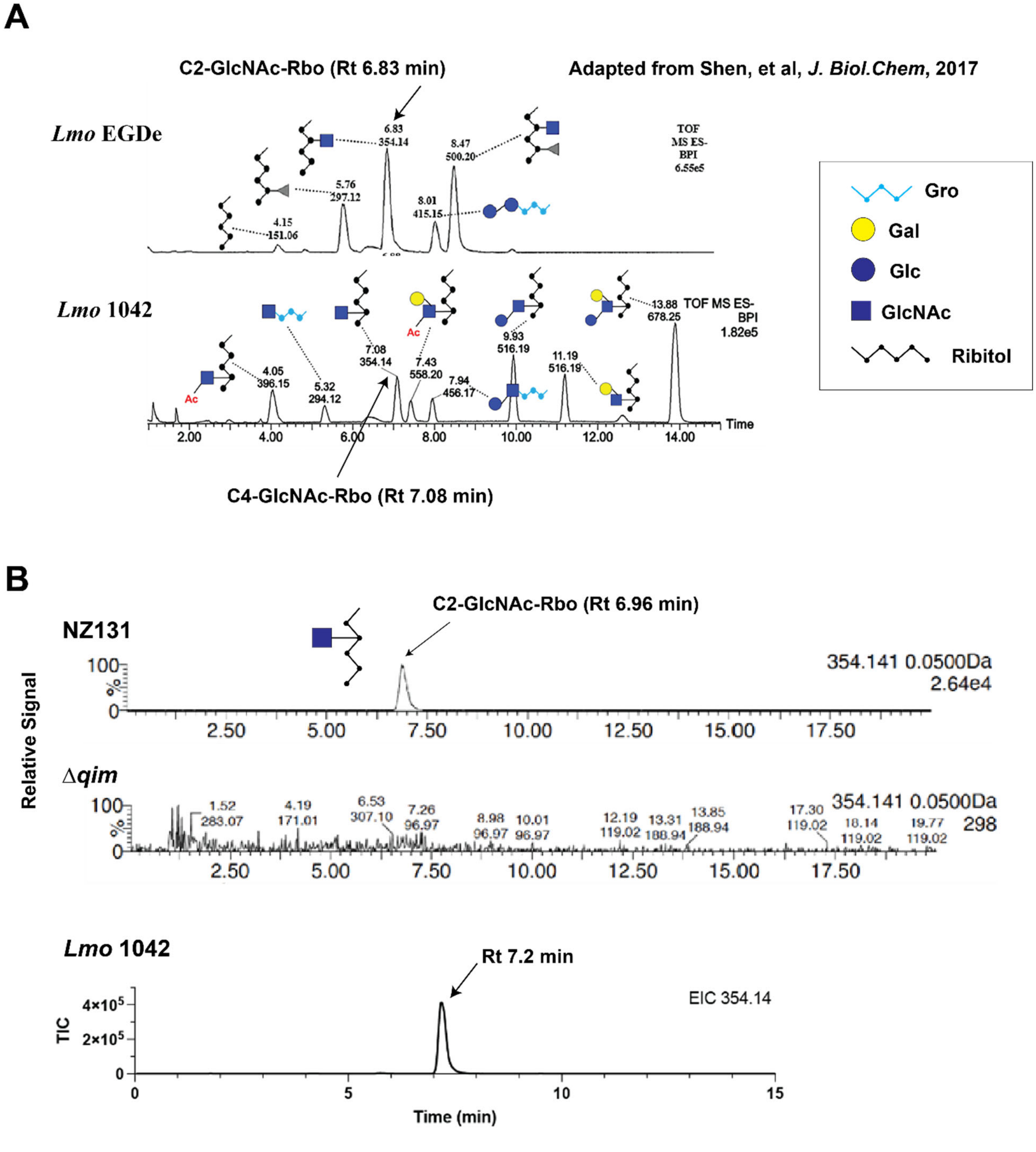


**Figure S8. (A)** UPLC-MS/MS monomer HF digestion analysis, showing representative ion chromatograms of reference samples *L. monocytogenes* EGDe (top) and 1042 (bottom). Indicated retention times of known GlcNAc-RboP (*m/z* 354.14) monomers showing C2- and C4-linkages, respectively. **(B)** QS-induced (ON) *S. pyogenes* NZ131 and Δ*qim* mutant showing extracted ion chromatograms (EIC) for m/z 354.14, compared to *Lmo*1042.

Supplemental Information

**Supplemental Table S1. Strains and plasmids used in this study.**

| **Strain** | **Description** | **Reference** |
|---|---|---|
| NZ131 | Wild-type M49 isolate | 1 |
| JCC303 | NZ131 Δ*spy49_0450-0460*; unmarked (Δ*qim*) | 2 |
| JCJ173 | NZ131 Δ*stcA*; unmarked | 3 |
| JCC303:pJC479 | NZ131 Δ*spy49_0450-0460*; attB::0450-0460 | This study |
| CMA101 | NZ131 Δ*spy49_0450-0460*; attB::0450-0460Δ0450 | This study |
| CMA102 | NZ131 Δ*spy49_0450-0460*; attB::0450-0460Δ0451 | This study |
| CMA105 | NZ131 Δ*spy49_0450-0460*; attB::0450-0460Δ0455 | This study |
| CMA106 | NZ131 Δ*spy49_0450-0460*; attB::0450-0460Δ0456 | This study |
| CMA107 | NZ131 Δ*spy49_0450-0460*; attB::0450-0460Δ0457 | This study |
| CMA108 | NZ131 Δ*spy49_0450-0460*; attB::0450-0460Δ0458 | This study |
| Δ*oatA* | NZ131 Δ*spy49_0035* | 3 |
| Δ*pgdA* | NZ131 Δ*spy49_1092c* | 3 |
| Δ*pplD* | NZ131 Δ*spy49_0642* | 4 |
| Δ*dltABCD* | NZ131 Δ*spy49_1034c-1037c* | 3 |
| Δ*gacH* | NZ131 Δ*spy49_0619* | 5 |
| **Plasmid** | **Description** | **Reference** |
| p7INT | Shuttle-suicide vector integrating at the streptococcal prophage tmRNA site attB; used for complementation in single copy; $erm^R$ | 6, 7 |
| pJC479 | Complementation of *spy49_0450-0460* in single copy under the constitutive syncat promoter; $erm^R$ | This study |
| pJC318 | Provided syncat promoter for construction of plasmid pJC479 | 8 |
| pCMA101 | To construct single gene deletion of *spy49_0450*; in pJC479; $erm^R$ | This study |
| pCMA102 | To construct single gene deletion of *spy49_0451*; in pJC479; $erm^R$ | This study |
| pCMA105_2 | To construct single gene deletion of *spy49_0455*; in pJC479; $erm^R$ | This study |
| pCMA106 | To construct single gene deletion of *spy49_0456*; in pJC479; $erm^R$ | This study |
| pCMA107 | To construct single gene deletion of *spy49_0457*; in pJC479; $erm^R$ | This study |
| pCMA108 | To construct single gene deletion of *spy49_0480*; in pJC479; $erm^R$ | This study |

erm = erythromycin

Table S1 references:


1. McShan, W. M. *et al*. Genome sequence of a nephritogenic and highly transformable M49 strain of Streptococcus pyogenes. *J. Bacteriol.* **190**, 7773-7785 (2008).
2. Rahbari KM, Chang JC, Federle MJ. A Streptococcus Quorum Sensing System Enables Suppression of Innate Immunity. mBio. 2021 Jun 29;12(3).
3. Gogos, A., Jimenez, J. C., Chang, J. C., Wilkening, R. V. & Federle, M. J. A quorum sensing-regulated protein binds cell-wall components and enhances lysozyme resistance in Streptococcus pyogenes. *J. Bacteriol.* (2018).
4. Rush JS, Parajuli P, Ruda A, Li J, Pohane AA, Zamakhaeva S, et al. PplD is a de-N-acetylase of the cell wall linkage unit of streptococcal rhamnopolysaccharides. Nat Commun. 2022 Feb 1;13(1):590.
5. Edgar RJ, van Hensbergen VP, Ruda A, Turner AG, Deng P, Le Breton Y, et al. Discovery of glycerol phosphate modification on streptococcal rhamnose polysaccharides. Nat Chem Biol. 2019 May 1;15(5):463–71.
6. Cho, K. H., Port, G. C. & Caparon, M. Genetics of Group A Streptococci. *Microbiol Spectr* **7** (2019).
7. McShan, W., McLaughlin, R., Nordstrand, A. & Ferretti, J. Vectors containing streptococcal bacteriophage integrases for site-specific gene insertion. *Methods Cell Sci* **20**, 51-57 (1998).
**8.** REF: DOI: 10.1128/jb.00176-22

**Supplemental Table S2. Primers used in this study.**

| Purpose | Primer | Sequence | Description |
|---|---|---|---|
| pJC479 | JC721 | AAGCATGCGGCCGCCCTCCTAAATTTTTATC | S primer for syncat promoter |
| | JC722 | CTTACACGGATCCAGATCTTGTCACCTAAATAG | AS primer for syncat promoter |
| | JC718 | AGGAGGGCGGCCGCATGCTTGTACTTTATG | S primer for first half of spy49_0450-0460 |
| | CMA100 | GTATAATGAAATTCCTTCTTTAAAATACTTTAATCTTCCATAAACTAAAATATTTCC | AS primer for first half of spy49_0450-0460 |
| | CMA101 | TTTAGTTTATGGAAGATTAAAGTATTTTAAAGAAGGAATTTCATTATACTACATTGG | S primer for second half of spy49_0450-0460 |
| | JC719 | AAGATCTGGATCCGTGTAAGTCAGATCAAC | AS primer for second half of spy49_0450-0460 |
| pCMA101 | CMA145 | GGGCGGCCGCATGACTTTTCATATTACAGTAAATCTTGATGAAATATCAGAC | S primer for iPCR; Δspy49_0450 |
| | CMA146 | GAAAAGTCATGCGGCCGCCCTCCTAAATTTTTATC | AS primer for iPCR; Δspy49_0450 |
| pCMA102 | CMA137 | CATGACGCGTAATGAATTTGAGTATATTTGG | S primer for iPCR; Δspy49_0451; MluI |
| | CMA138 | CATGACGCGTTCATTTTCCTTTTATCTTCCC | AS primer for iPCR; Δspy49_0451; MluI |
| pCMA105_2 | JC732 | CATGACGCGTTAAAACCAATGTTTATGATGG | S primer for iPCR; Δspy49_0455; MluI |
| | JC733 | CATGACGCGTTAAATCCATAGTTACACCTC | AS primer for iPCR; Δspy49_0455; MluI |
| pCMA106 | CMA141 | CATGACGCGTGAAAAACTCATAGTGTGGG | S primer for iPCR; Δspy49_0456; MluI |
| | CMA142 | CATGACGCGTTTTATCCATCATAAACATTGG | AS primer for iPCR; Δspy49_0466; MluI |
| pCMA107 | JC734 | CATGACGCGTTCTTACTCCTTTTTCAATACTTC | S primer for iPCR; Δspy49_0457; MluI |
| | JC735 | CATGACGCGTGAGAAGAAGTGTGATTGGTGG | AS primer for iPCR; Δspy49_0457; MluI |
| pCMA108 | CMA151 | GTACAACTAATTTCTAACAAAAAAATTGATTTATAAAAAGTTTTAG | S primer for iPCR; Δspy49_0458 |
| | CMA152 | TTGTTAGAAATTAGTTGTACTTTAGATAATTGTATACTC | AS primer for iPCR; Δspy49_0458 |

**TABLE S3.** NMR CHEMICAL SHIFTS ($^{1}$H AND $^{13}$C) OF GROUP A CARBOHYDRATE (WILDTYPE QS-ON) FROM $^{1}$H,$^{13}$C-HSQC EXPERIMENT[a]

| Monomer | $^{1}$H/$^{13}$C HSQC | | | | | | |
|---|---|---|---|---|---|---|---|
| | 1 | 2 | 3 | 4 | 5 | 6 | Me |
| **-3)-α-L-Rha*p*-(1-2)** | 5.11<br>100.94 | 4.30<br>76.41 | 4.02<br>79.63 | 3.54<br>71.32 | 3.84<br>69.37 | 1.33<br>16.97 | |
| **-2,3)- α-L-Rha*p*-(1-3)** | 5.05<br>102.21 | 4.09<br>69.86 | 3.87<br>76.90 | 3.57<br>71.81 | 3.75<br>69.37 | 1.28<br>16.68 | |
| **β-D-Glc*p*NAc-(1-3)** | 4.76<br>102.51 | 3.75<br>55.98 | 3.57<br>73.96 | 3.47<br>69.86 | 3.48<br>75.82 | 3.79, 3.96<br>(4.12, 4.19)<br>60.86 (64.38) | 2.05<br>22.35 |
| **L-Gro-1-*P*** | 3.91, 3.96<br>66.44 | 3.94<br>70.74 | 3.65, 3.71<br>62.13 | | | | |

[a] referenced against and matching the reported values by Edgar *et al*., 2019.

Edgar RJ, van Hensbergen VP, Ruda A, Turner AG, Deng P, Le Breton Y, et al. Discovery of glycerol phosphate modification on streptococcal rhamnose polysaccharides. Nat Chem Biol. 2019 May 1;15(5):463–71.
